# The Effect of *Gynostemma pentaphyllum* Extract on Mouse Dermal Fibroblasts

**DOI:** 10.1155/2014/202876

**Published:** 2014-03-04

**Authors:** Sara Nadia Lobo, Yu Qing Qi, Quan Zhong Liu

**Affiliations:** Department of Dermatology, Tianjin Medical University General Hospital, International Students' Building, Tianjin Medical University, Qixiangtai Road, No. 22, Heping District, Tianjin 300070, China

## Abstract

*Background.* The objective of this paper is to demonstrate the effect of *Gynostemma pentaphyllum* extract on mouse dermal fibroblasts. Recent studies have shown that this plant may possess great antioxidant properties, which can be very beneficial in combating oxidative stress. *Methods. Gynostemma pentaphyllum* extract was prepared and mouse dermal fibroblasts were obtained and cultured as per our laboratory protocols. Twelve samples of cells were cultured under the same conditions and both negative and positive controls were established. Induction of oxidative stress was carried out using ultraviolet C (UVC) light. Viable cell count was carried out, using microscopy. The analysis of the overall results was processed using SPSS version 16.0. *Results.* Statistical analysis showed strong positive correlation between the concentration of *Gynostemma pentaphyllum* and the mean duration of cell viability (rs = 1), with a high level of statistical significance (*P* < 0.01). Likewise, strong positive correlation existed between trials of cell viability (rs = 0.988–1), with statistical significance (*P* < 0.01). *Conclusion. Gynostemma pentaphyllum* extract prolongs viability of mouse dermal fibroblasts damaged by UVC light-induced oxidative stress. The results show the potential benefits of this extract on dermal cell aging.

## 1. Introduction

The retardation and control of skin aging constitute one of the biggest challenges faced by researchers and scientists in the area of cosmetology. This area of study is continuously researched, due to the unending pursuit of maintenance of youthfulness and appearance. Many scientific investigations are currently underway, as the field of cosmeceutical development widens. New studies are revealing the truth about many agents that modify the process of skin aging. The future incorporation of these studies into clinical practice would change the way that this process is currently managed.

Many plant extracts play a vital role in the modification of skin aging. Some of these have been thoroughly investigated, whereas others have only been used empirically for centuries. *Gynostemma pentaphyllum*, commonly referred to as “Jiaogulan” in China, is one of the many examples of these plants [1, 2].

This plant is normally grown in the mountainous regions of China and is considered a vine because of its growth pattern. It has been used for centuries as a traditional remedy for respiratory and toxic conditions and has also been associated with longevity, among other benefits [1, 2].

Many studies have been done to verify some of the many beliefs associated with the use of this herb; however, as per the extensive research done prior to this proposal, no studies regarding the action of the plant extract on dermal fibroblasts have been documented or published.

The objective of this paper is to study the effects of *Gynostemma pentaphyllum* extract on dermal fibroblast, as it relates to skin aging. The hypothesis of this study is directed in favor of this plant extract, as being a significant contributor to the process of retardation of skin aging. This study has great scientific significance and will further contribute to the complete understanding of the benefits of this herb. This paper opens up the possibility for the development of scientifically based cosmeceutical products, designed to combat the signs of skin aging.

## 2. Materials and Methods

1. Preparation of *Gynostemma pentaphyllum* extract: 100 mL of double distilled water was heated to boiling point (100°C). 100 mg of the *Gynostemma pentaphyllum* herb was macerated and added to the double distilled water at boiling point. The mixture was then allowed to cool to room temperature. The solution was sterilized with 0.22 micrometer filters, sealed and subsequently stored at a 4˚C in a refrigerator. A variety of different concentrations of the extract were subsequently obtained from the original solution.
2. Mouse dermal fibroblasts were obtained and cultured as per protocols established by our Laboratory of the Department of Dermatology of the Tianjin Medical University General Hospital. Twelve samples of cells were cultured under the same conditions. One of the twelve samples was used as the positive control and was kept under optimum conditions throughout the entire experiment, without exposure to any aggressions or stimulants.
3. The other eleven samples were placed under 8 watts—ultraviolet C (UVC) light at a distance of 50 cm to induce oxidative stress. Induction of oxidative stress was limited to 1.5 hours.
4. Subsequently, one of the eleven samples was placed under optimum conditions throughout the rest of the experiment, without any further exposure to aggressions or stimulants. This sample was considered as the negative control.
5. The remaining ten samples were exposed to similar quantities of different concentrations of the *Gynostemma pentaphyllum* extract and, as with the rest of the samples, they were correctly labeled and placed under optimum conditions throughout the rest of the experiment, without any further exposure to aggressions or stimulants.
6. Qualitative analysis of cell viability was carried out using microscopy, as per our laboratory standards, at zero hours, followed by subsequent intervals.
7. The entire procedure was officially repeated twice, for a total of three trials.
8. Results obtained from the periodic observations were carefully and accurately documented.
9. The analysis of the overall results was processed using SPSS version 16.0. Spearman's correlation coefficient was used to demonstrate the relationship between the variables used.
10. The results were presented using charts and tables.

## 3. Results

**Figure 1:**
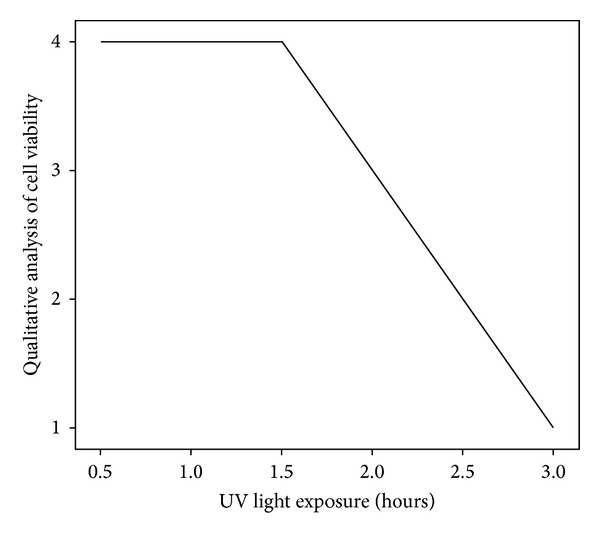
Relationship between cell viability and exposure to UVC light. For qualitative analysis of cell viability: 4—all cells viable; 3—more viable cells than apoptotic cells; 2—more apoptotic cells than viable cells; 1—total apoptosis.

**Figure 2:**
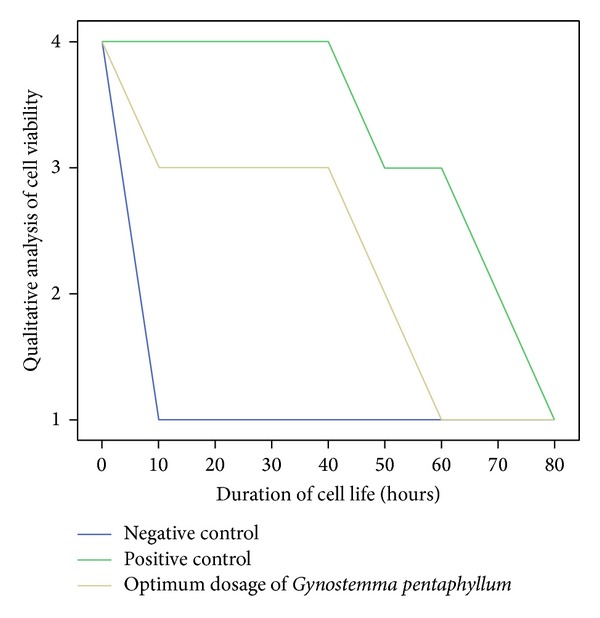
Relationship between cell samples and the duration of cell life. For qualitative analysis of cell viability: 4—all cells viable; 3—more viable cells than apoptotic cells; 2—more apoptotic cells than viable cells; 1—total apoptosis.

**Figure 3:**
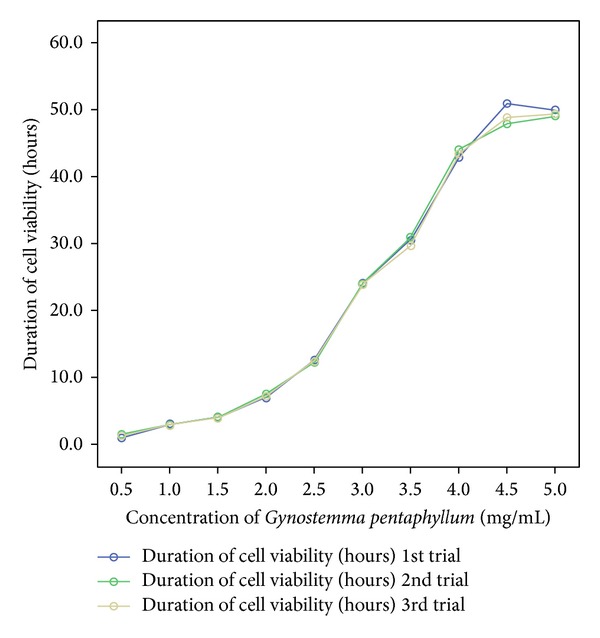
Relationship between cell viability and exposure to *Gynostemma pentaphyllum* following UVC light-induced oxidative stress.

**Figure 4:**
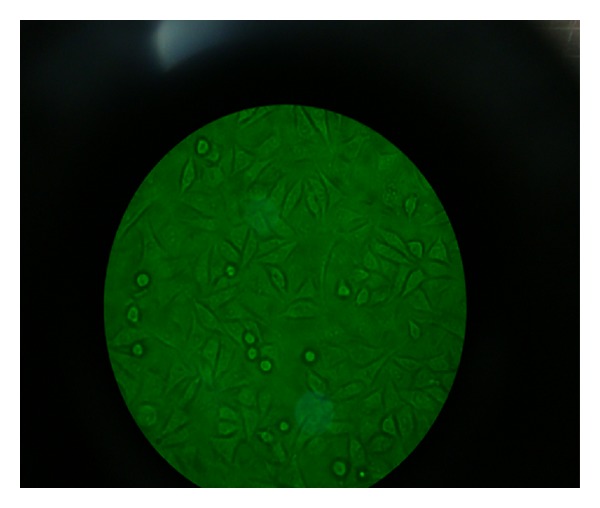
Photographs of various stages of the investigation (magnification 40x, no stain used). All cells viable.

**Figure 5:**
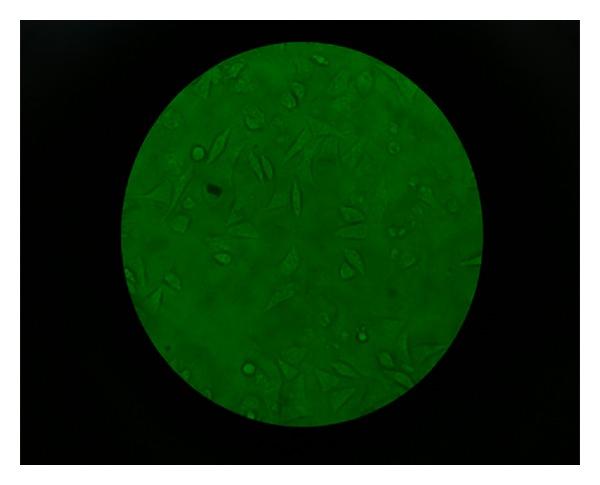
Photographs of various stages of the investigation (magnification 40x, no stain used). More viable cells than apoptotic cells.

**Figure 6:**
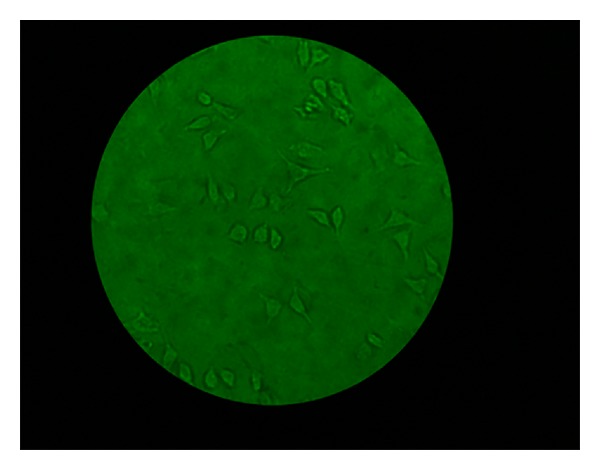
Photographs of various stages of the investigation (magnification 40x, no stain used). More apoptotic cells than viable cells.

**Figure 7:**
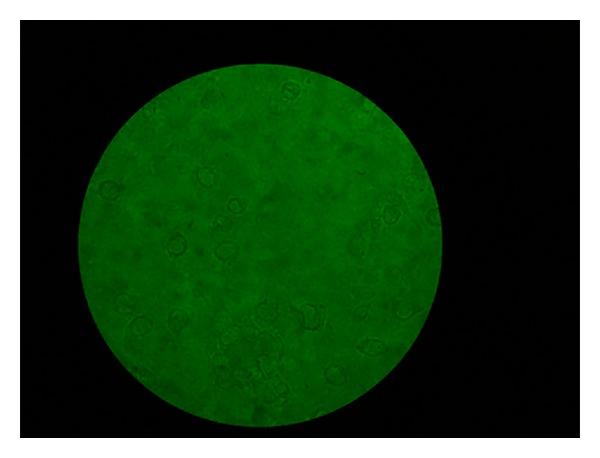
Photographs of various stages of the investigation (magnification 40x, no stain used). Total apoptosis.

UVC light induced a maximum level of oxidative stress at 1.5 hours of exposure (See Table 1 and Figure 1). After this period, signs of apoptosis were clearly visible (See Figures 4, 5, 6, and 7). Qualitative analysis of cell viability revealed that an optimum concentration of *Gynostemma pentaphyllum* (taken as 4.5 mg/mL) significantly prolonged the viability of cells exposed to UVC light (See Table 2 and Figures 2 and 3). Statistical analysis showed strong positive correlation between the concentration of *Gynostemma pentaphyllum* and the mean duration of cell viability (rs = 1) with a high level of statistical significance (*P* < 0.01) (See Table 3(a)). Likewise, strong positive correlation existed between trials of cell viability (rs = 0.988–1) with statistical significance (*P* < 0.01) (See Table 3(b)).

## 4. Discussion

*Gynostemma pentaphyllum* belongs to the family of Cucurbitaceae and has been associated with the treatment of cancer, gastric ulcers, hepatitis, respiratory diseases, and hyperlipidaemia, among other pathologies. One of the most recently discovered benefits of this herb is its antioxidant properties [1, 2].

The exact mechanisms involved in the pathophysiology of skin aging indicate that oxidative stress plays a vital role in the progress of this process. Any measures that can be taken to suppress the production of free radicals will facilitate the preservation of youthfulness of the skin. After thorough research on some of the naturally occurring antioxidants, *Gynostemma pentaphyllum* appears to be one of the most promising. Our results indicate that *Gynostemma pentaphyllum* is effective in prolonging the life of mouse dermal fibroblasts and that this effect increases in a dose-dependent manner.

Many studies have been done to assert the potential of *Gynostemma pentaphyllum* as an antioxidant. The cells used in most of the studies vary widely, but the results are all statistically significant. The action of this plant extract on dermal fibroblasts has not been investigated, as per the research and review done. No studies are currently available to verify the effect of this extract on dermal fibroblast.

Schild et al. demonstrated the protective action of *Gynostemma pentaphyllum* extract on brain slices that were deprived of oxygen and glucose. This deprivation led to the onset of cell injury due to oxidative stress. Results showed that the use of this extract, within 48 hours of cultivation of cells, increased protein and activity levels of the antioxidative enzymes manganese superoxide dismutase (Mn-SOD) and glutathione peroxidase (GPx). Consequently, the cellular hydrogen peroxide (H_2_O_2_) concentration remained at a low level. Similar antioxidant effects have been described in the findings of the study carried out by Zhang et al., who asserted the antioxidant properties of *Gynostemma pentaphyllum* extract on brain cells [3, 4]. Likewise, significant favorable results have been obtained by different researchers who also investigated the protective antioxidant effect of *Gynostemma pentaphyllum* on brain cells [5, 6]. Our results are consistent with those of the authors aforementioned, and these results validate the effectiveness of this extract in combating oxidative stress.

Müller et al. further contributed to studies regarding the antioxidant potential of this herb extract. The study was carried out using hepatocytes that were exposed to substances that induced oxidative stress. The extract completely protected the cells from oxidative stress-induced apoptosis. Similar results have been obtained by Lin et al. in their investigation using hepatocytes [7, 8].

Other studies done, using phagocytes and endothelial cells, such as that of Li et al., have produced significant results [9]. The fact that the action of this extract is nonspecific created the base for pursuing our investigation. Our results now reveal that this extract is effective in prolonging the life of mouse dermal fibroblasts exposed to UVC light-induced oxidative stress.

Research done by Ma and Yang, at the Graduate School of the University of Science and Technology of Beijing demonstrated that *Gynostemma pentaphyllum* could scavenge active oxygen free radicals effectively [10]. Zhu et al. demonstrated in their study, using UV radiation, that *Gynostemma pentaphyllum* not only is a scavenger of free radicals but also possesses the biological function of anti-irradiation [11].

Other advantages of *Gynostemma pentaphyllum* have been discovered by other researchers. Gauhar et al. investigated the effect of *Gynostemma pentaphyllum* extract on obesity. This study was carried out at the preclinical level and has demonstrated significant results. The molecular mechanism of action involved the stimulation of fat oxidation and glucose uptake via AMPK activation in L6 myotube cells, with a corresponding decrease in body weight gain, liver weight, and blood cholesterol levels in mice [12].

In summary, our study shows that *Gynostemma pentaphyllum* is capable of reducing the effects of oxidative stress on mouse dermal fibroblasts. Oxidative stress accelerates aging and apoptosis. In our study, the prolongation of cell viability following UV induced oxidative stress served as a parameter for inferring that *Gynostemma pentaphyllum* extract acted as an antioxidant. This extract may significantly reduce the effects of oxidative stress on human dermal fibroblasts and may also reduce the aging and apoptosis of these cells. We carried out a qualitative assessment of mouse dermal fibroblast viability after exposure to UVC, in the presence and absence of *Gynostemma pentaphyllum* extract, as an initial phase in our ongoing research. Since no initial published results were available on this topic, we commenced our research with a qualitative, *in vitro* study. Given the fact that our results are promising, our team would proceed to perform a quantitative, preclinical study to corroborate our initial results. More research and investigation are required on this topic, in order to gain more insight into the actual mechanism of action of the extract on dermal cells. Our study also serves as a foundation on which new cosmeceutical products, such as antiaging and antioxidant creams and lotions, can be developed.

## 5. Conclusion

Our study reveals that *Gynostemma pentaphyllum* extract has a positive effect on the viability of mouse dermal fibroblasts that have been damaged by UVC light-induced oxidative stress. There is a very high level of association between the use of *Gynostemma pentaphyllum* and the prolonging of cell life. The potential repercussions of such an investigation are clearly visible, since this investigation creates a foundation for further studies in this field of dermatology. In addition, more profound research is required on this particular topic in order to establish valid comparisons between the results obtained and to isolate the actual compounds that are responsible for protecting the cells.

## Figures and Tables

**Table 1:** Relationship between cell viability and exposure to UVC light.

UV light exposure *t*/hrs	Qualitative analysis of cell viability
0	++++
0.5	++++
1	++++
1.5	++++
2	+++
2.5	++
3	+

L ++++: all cells viable; +++: more viable cells than apoptotic cells; ++: more apoptotic cells than viable cells; +: total apoptosis.

**Table 2:** Relationship between cell samples and duration of cell life.

Duration of cell life/hrs	Negative control	Positive control	Optimum concentration of *Gynostemma pentaphyllum *
0	++++	++++	++++
10	+	++++	+++
20	+	++++	+++
30	+	++++	+++
40	+	++++	+++
50	+	+++	++
60	+	+++	+
70	+	++	+
80	+	+	+

L ++++: all cells viable; +++: more viable cells than apoptotic cells; ++: more apoptotic cells than viable cells; +: total apoptosis.

**Table:** (a)

		Concentration of *Gynostemma pentaphyllum* (mg/mL)	Mean duration of cell viability
Spearman's rho			
Concentration of *Gynostemma pentaphyllum* (mg/mL)	Correlation coefficient	1.000	1.000**
	Sig. (2-tailed)	—	—
Mean duration of cell viability	Correlation coefficient	1.000**	1.000
	Sig. (2-tailed)	—	—

**Correlation is significant at the 0.01 level (2-tailed).

**Table:** (b)

		Duration of cell viability (hours) 1st trial	Duration of cell viability (hours) 2nd trial	Duration of cell viability (hours) 3rd trial
Spearman's rho				
Duration of cell viability (hours) 1st trial	Correlation coefficient	1.000	0.988**	0.988**
	Sig. (2-tailed)	—	0.000	0.000
Duration of cell viability (hours) 2nd trial	Correlation coefficient	0.988**	1.000	1.000**
	Sig. (2-tailed)	0.000	—	—
Duration of cell viability (hours) 3rd trial	Correlation coefficient	0.988**	1.000**	1.000
	Sig. (2-tailed)	0.000	—	—

**Correlation is significant at the 0.01 level (2-tailed).
